# Decreased glycolysis induced dysfunction of NK cells in Henoch-Schonlein purpura patients

**DOI:** 10.1186/s12865-020-00382-9

**Published:** 2020-10-09

**Authors:** Wenjia Chai, Xiaolin Wang, Wei Wang, Hui Wang, Wenjun Mou, Jingang Gui

**Affiliations:** Laboratory of Tumor Immunology, Beijing Pediatric Research Institute, Beijing Children’s Hospital, Capital Medical University, National Center for Children’s Health, Beijing, 100045 China

**Keywords:** Henoch-Schonlein purpura, Henoch-Schonlein purpura nephritis, NK cells, Glycolysis, Metabolic reprogramming

## Abstract

**Background:**

Henoch-Schonlein purpura (HSP) is the most common systemic vasculitis of the childhood. However, its mechanisms and pathogenesis still need more exploration. Natural killer (NK) cells are innate lymphocytes, and there is a growing appreciation that cellular metabolism is important in determining the immune responsiveness of lymphocytes. Thus, we aimed to analyze the NK cells phenotype and explore the association between glucose metabolism and NK cells function in HSP patients.

**Results:**

A total number of 64 HSP patients and 34 healthy children were included. The HSP patients were divided into two groups according to whether accompanied with nephritis or not. NK cells in HSP patients without nephritis showed a reduced frequency in peripheral blood, a down-regulated expression of activating receptors both NKp30 and NKp46, and an attenuated cytotoxic function against tumor cells. In addition, the function impairment of NK cells was shown to exacerbate in HSPN. Our data further revealed an aberrant metabolic reprogramming of NK cells in HSP patients. Upon stimulation with cytokines (IL-15, IL-12 and IL-2), NK cells from healthy controls switched to an elevated glycolysis rate to support their effector function. By contrast, the glycolysis rate of activated NK cells in HSP group was not significantly up-regulated from the resting level possibly owing to the inhibition of mTORC1.

**Conclusions:**

Our study found that HSP patients were accompanied with dysfunction of NK cells. We concluded that the dysfunction of NK cells in HSP patients was induced with a decreased glycolysis rate and suggested that metabolic reprogramming of NK cells might be a player in the pathogenesis of HSP.

## Background

Henoch-Schonlein purpura (HSP), also referred to as IgA vasculitis, is characterized by immunoglobulin A1 (IgA1)-dominant immune deposits affecting small vessels [1]. The multiple manifestations of the disease include nonthrombocytopenic purpura, arthritis, gastrointestinal involvement and nephritis [2]. Among HSP nephritis (HSPN) patients, most possess a mild form of the disease, presenting with only hematuria and/or low-grade proteinuria, while a few cases will develop into nephrotic syndrome or renal function impairment [2, 3]. The severity of renal involvement was proved to affect the disease prognosis greatly [4]. HSP is frequently reported to follow respiratory infections, and a variety of pathogens, such as viral and bacterial pathogens, have been implicated as triggers of the disease [5, 6]. Although the aetiology of HSP is not well-known, there are some evidence to support immunopathological mechanisms [7]. The clinical manifestation of HSP is the consequence of a leukocytoclastic vasculitis, which frequently occurs following an infectious trigger and involves IgA1 and C3 deposition in small vessel walls [5]. The diminished glycosylation of the hinge region of IgA1 in HSP patients are prone to aggregate into macromolecular complexes, which activate the pathway of complement and deposit affected organs [8, 9]. Several inflammatory cytokines, such as IL-17, IL-8, IL-6 and TGF-α, were involved in IgA elevation in HSP patients [10–13]. In addition, immune cells such as Th1, Th2, Th17 and Treg cells also participate in the progress of HSP [14–16], but the role of NK cells in HSP or HSPN pathogenesis were undefined.

Natural killer (NK) cells are important innate effector cells that directly kill tumor and pathogen-infected cells [17]. Resting NK cells patrol in the blood and infiltrate into tissues to exert effector functions once activated [18]. Previous studies showed that NK cells were involved in IgA nephropathy (IgAN), which is also related to the abnormal IgA1 glycosylation and deposition [19]. However, the role of NK cells in IgAN is still controversial. For example, in IgAN patients, NK cells exerted cytotoxic activity toward human glomerular endothelial cells, which seemed to be responsible for hematuria [20]. Nevertheless, NK cells were proved to be able to enhance IgA synthesis from B cells [21]. It has been demonstrated that IgA bound to Fc receptors on NK cells surface and mediated the inhibition of cytotoxic effect [22, 23]. Given that HSP is mediated by an antigen-stimulated IgA and deposition of IgA-containing immune complexes, we hypothesized that NK cells may be related to the onset, course or prognosis of HSP.

The activation of NK cells is driven by a balance between activating and inhibitory signals from various cytokines interaction and NK cell receptors (NKR) ligation without a requirement for previous sensitization [24]. There is a growing appreciation that cellular metabolism is important in determining the immune responsiveness of lymphocytes [25]. Upon immune activation, lymphocytes, including NK cells, reprogram the cellular metabolism to aerobic glycolysis, by which an enhanced glycolysis metabolizing much glucose into lactate is achieved [26–28]. Recent studies have demonstrated that resting NK cells depended mainly on OXPHOS for their survival [26, 29]. When activated by cytokines or NKR stimulation, NK cells promoted both glycolysis and OXPHOS to support IFN-γ production [27, 30]. In addition, several studies revealed that the deviant alteration in glycolysis have been linked to NK cells dysfunction. For instance, inhibition of glycolysis decreased NK cell killing, dampened NK cell degranulation and Fas ligand expression, suggesting that glycolysis is critical for proper function of cell cytotoxicity [30]. Recent studies revealed that the metabolic profile of NK cells was different under certain conditions, such as obesity [31, 32] or during viral infection [33]. However, there are few studies on glucose metabolism in HSP patients and its relationship with HSP pathogenesis has not yet been illuminated.

Thus, we aimed to analyze the NK cells phenotype and explore the association between glucose metabolism and NK cells function of HSP patients.

## Results

### Frequencies of NK cells were reduced in HSP patients

**Fig. 1:**
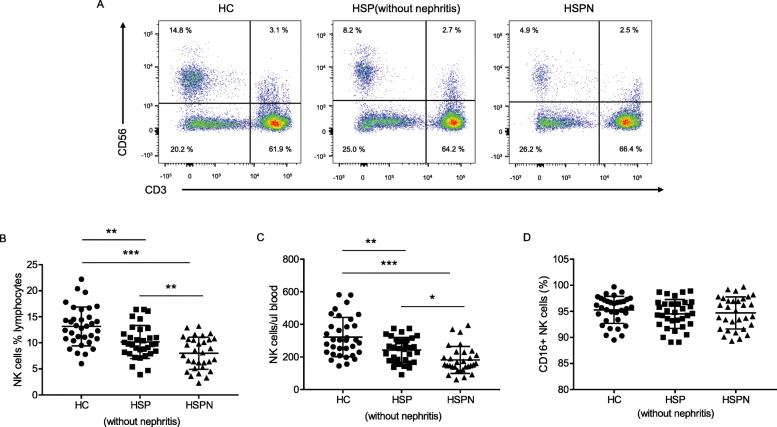
Frequencies of NK cells were reduced in HSP patients. **a** Representative dot plots showed the percentage of CD56+/CD3- NK cell in peripheral blood. **b** Scatter plots showed NK cell frequencies of the indicated groups. **c** Scatter plots showed the absolute number of NK cell from indicated groups. **d** The frequencies of CD16+ NK cell were shown. HC, healthy controls, *n* = 34; HSP (without nephritis), *n* = 33; HSPN, HSP patients with nephritis, *n* = 31. *p < 0.05, **p < 0.01, and ***p < 0.001 (one-way ANOVA and Tukey’s multiple comparisons test)

We initially investigated the frequencies and absolute counts of CD3+ T lymphocytes subset, CD19+ B cells and CD56+/CD3- NK cells from each group (aged from 3 to16, clinical information described in Table 1). The statistical results and representative flow plots were displayed (Table 2, Fig. 1a). Consistent with previous study, the ratio of CD4/CD8 was decreased in HSP patients [34]. The percentage of CD19+ B cells showed reduction, while absolute count had no significant difference. The multiple analysis indicated that the frequency and absolute count of CD56+ CD3- NK cells in HSP patients were decreased compared with that in healthy controls, and further reduced in HSPN patients (Fig. 1 a, b, c). We also examined the proportion of CD16+ NK cells, a subpopulation with cytotoxic property [35], and no significant difference was observed (Fig. 1c). In other words, a biased drop in specific NK subpopulations was not observed and was not associated with deteriorated NK reduction in HSP patients with renal damage.

**Table 1:** Patient information

Group	Gender	Age	Involvement parts
Healthy controls *n* = 34	Boy (54.5%) Girl (43.5%)	9.12 ± 0.48	–
HSP group (HSP without nephritis) *n* = 33	Boy (54.5%) Girl (45.5%)	8.70 ± 0.60	Skin (100%) Gastrointestinal tract (58.8%) Joints (20.6%) Kidney (0%)
HSPN group (HSP with nephritis) *n* = 31	Boy (52.9%) Girl (47.1%)	9.42 ± 0.60	Skin (100%) Gastrointestinal tract (61.3%) Joints (19.4%) Kidney (100%)

**Table 2:** The frequencies and absolute counts of T lymphocytes subset, B cells and NK cells from each group [Mean ± SEM]

Parameters	HC(*n* = 34)		HSP without nephritis (*n* = 33)		HSPN (*n* = 31)	
	% lymphocytes	Counts (cells/ul)	% lymphocytes	Counts (cells/ul)	% lymphocytes	Counts (cells/ul)
CD3+ T cell	66.6 ± 4.2	1631.8 ± 390.0	66.4 ± 4.8	1581.2 ± 383.2	68.9 ± 4.9	1722.4 ± 868.2
CD4+ T cell	34.0 ± 3.3	829.7 ± 191.5	32.7 ± 3.8	783.6 ± 210.5	32.4 ± 3.9	825.6 ± 436.6
CD8+ T cell	25.5 ± 8.8	610.1 ± 166.7	27.4 ± 6.4	656.0 ± 221.1	30.3 ± 4.6	735.8 ± 332.0
B cell	16.4 ± 3.6	404.8 ± 147.0	19.0 ± 4.5	456.9 ± 159.3	19.4 ± 4.3	506.1 ± 365.1
NK cell	13.1 ± 3.7	322.4 ± 120.8	10.2 ± 3.2	242.5 ± 77.1	8.0 ± 3.1	181.4 ± 82.8

### NKp30 and NKp46 exhibited a down-regulation in HSP NK cells

**Fig. 2:**
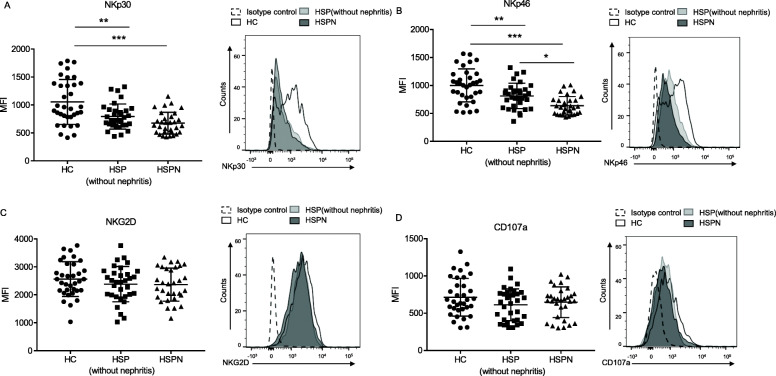
NKp30 and NKp46 showed down-regulation of HSP patients, especially HSPN patients. (**a** to **d**) Phenotypes of NK cells were analyzed by flow cytometry. The mean fluorescence intensity (MFI) and representative histogram of NKp30, NKp46, NKG2D and CD107A were shown from A to D. HC, *n* = 34; HSP (without nephritis), *n* = 33; HSPN, *n* = 31. *p < 0.05, **p < 0.01, and ***p < 0.001 (one-way ANOVA and Tukey’s multiple comparisons test)

Considering the differential percentage of circulating NK cells between patients and healthy controls, we intended to explore if there was any aberrant activation in HSP patients. We found NKp30 and NKp46, two activating receptors expressing on NK cells, were down-regulated. However, the expression of another activating receptor NKG2D and the degranulation marker CD107a showed no significant difference compared with controls. What’s more, we found the expression of NKp46 on NK cells from HSPN patients was further decreased compared with HSP patients without renal damage (Fig. 2 a, b).

Overall, our data suggested that the activation of NK cells was partially impaired in HSP patients, and it was more severe in those with renal damage.

### NK cells function was impaired in HSP and HSPN patients

**Fig. 3:**
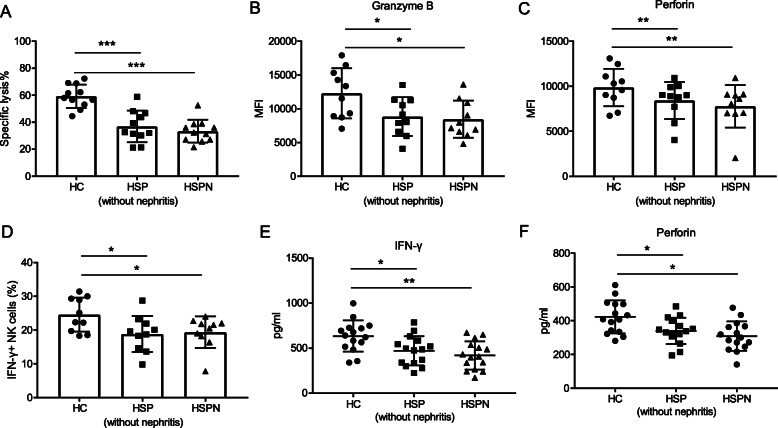
NK cells function was impaired in HSP and HSPN patients (**a**) NK cells from indicated groups were co-incubated with K562 target cells for 6 h at 10: 1 effecter/target ratio. The percentage of K562 cells lysed were analyzed. **b**, **c** The MFI of perforin and granzyme B were determined by flow cytometry. **d** The percentage of IFN-γ + NK cells were shown. Results were expressed as mean ± SEM. *n* = 10, *p < 0.05, **p < 0.01, and ***p < 0.001 (one-way ANOVA and Tukey’s multiple comparisonsStudent’s t test). **e**, **f** Isolated NK cells were treated with IL-15 (10 ng/ml), IL-2 (20 ng/ml) and IL-12 (30 ng/ml) for 18 h. The supernatants were collected and assayed for IFN-γ and perforin by ELISA. Results were expressed as mean ± SEM. *n* = 15, *p < 0.05, **p < 0.01, and ***p < 0.001 (one-way ANOVA and Tukey’s multiple comparisons)

Considering that NK cell activation in HSP patients was impaired, we investigated the cytotoxic function of NK cells. For this aim, 10 candidates were randomly selected for following studies. The isolated NK cells were firstly stimulated with cytokines (IL-15, IL-12 and IL-2) for 7 days and their cytotoxic function was then evaluated via a 6-h co-culture with a NK sensitive tumor cell line, K562. The percentage of NK-lysed K562 cells was determined by LDH release to the supernatant. The results showed that the NK cells from HSP and HSPN patients killed fewer target K562 cells compared with healthy children (Fig. 3 a). Parallel to this observation, both intracellular molecules granzyme B and perforin showed diminished expression in HSP and HSPN NK cells (Fig. 3 b, c). In addition, the effector cytokines, IFN-γ, showed a significant reduction (Fig. 3 d). The dysfunction of NK cells was further confirmed by the ELISA analysis about the concentration of perforin and IFN-γ in serum (Fig. 3 e, f). These results implied that the cytotoxic function of NK cells was impaired in HSP patients.

### The activated NK cells from HSP patients possessed a decreased glycolysis rate

**Fig. 4:**
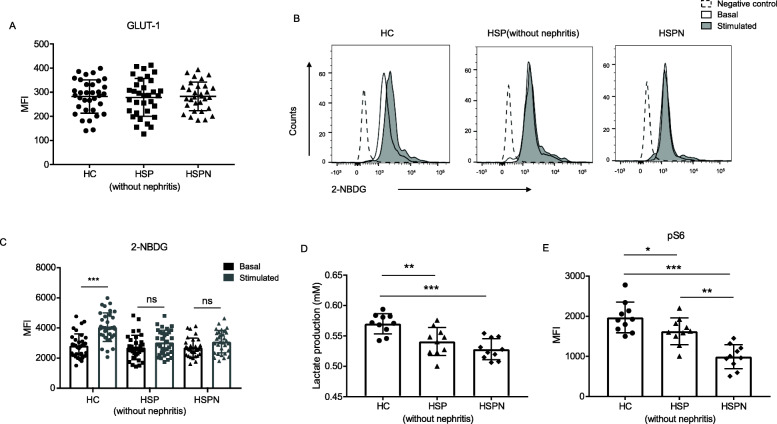
NK cells from HSP patients, especially HSPN patients, displayed decreased glucose uptake and lactate production. **a** Scatter plot showed the Glut-1 expression (MFI) of NK cells. HC, *n* = 34; HSP (without nephritis), *n* = 33; HSPN, *n* = 31. **b**, **c** The NK cells were treated with IL-15 (10 ng/ml), IL-2 (20 ng/ml) and IL-12 (30 ng/ml) for 18 h. Representative histogram and bar graphs showed the 2-NBDG uptake of basal and stimulated NK cells of indicated groups. HC, *n* = 34; HSP (without nephritis), *n* = 33; HSPN, *n* = 31. *p < 0.05, **p < 0.01, and ***p < 0.001 (Student’s *t* test). **d** Lactate production of indicated groups of activated NK cells were investigated. Results were expressed as mean ± SEM. *n* = 10, *p < 0.05, **p < 0.01, and ***p < 0.001 (one-way ANOVA and Tukey’s multiple comparisons). (E) Expression of phosphorylation of ribosomal S6 (pS6) of activated NK cells were measured with flow cytometry. Results were expressed as mean ± SEM. *n* = 10, *p < 0.05, **p < 0.01, and ***p < 0.001 (one-way ANOVA and Tukey’s multiple comparisons)

The function of effector NK cells indispensably rely on metabolic reprogramming. Once activated, NK cells will switch the balance of the core metabolic program from oxidative phosphorylation (OXPHOS) to glycolysis, aiming to meet the increased energy requirement to perform normal immune function [36]. As NK cells from HSP patients displayed abnormality in terms of activating receptor expression and cytotoxic function, we explored whether the glycolysis rate of NK cells was different from healthy controls. Glucose is a major energy substrate to generate adenosine triphosphate (ATP) for multiple cellular processes to support cell functions [37]. Therefore, we examined the expression of Glut-1, a major glucose transporter on NK cells and found no significant difference among HSP, HSPN and healthy groups (Fig. 4a).

We next assessed the glucose uptake capacity of NK cells with 2-NBDG, a fluorescent glucose analog. Our results showed that the basal glucose consumption of NK cells from the three groups were comparable. However, upon cytokines stimulation, we observed an increased 2-NBDG uptake by NK cells from healthy children but not from HSP groups, heedless of the presence of nephritis or not (Fig. 4b, c). In addition, compared with HSP patients, HSPN patients possessed a reduced glucose uptake capacity, which was reflected by the slightly lower average intracellular 2-NBDG fluorescence intensity. (Fig. 4b, c). Lactate release is a hallmark of metabolic glycolysis activity of cells [38]. The lactate concentrations determined from isolated NK cells subjected to cytokine stimulation exhibited a remarkably decreased level in HSP patients (Fig. 4d). As mTOR sensing nutrients and growth factors and functioning for metabolic reprogramming of NK cells [39], we next studied the activation of mTORC1 via the down-stream phosphorylation of S6. Following 18 h stimulation, mTORC1 activity of HSP NK cells were significantly inhibited, and a noticeable further down-slope change in HSPN group was secured (Fig. 4e). Together, these results indicated that the glucose uptake capacity and the glycolysis rate of activated NK cells from HSP patients were significantly decreased, which might be a consequence from the inhibition of mTORC1. In addition, NK cells from HSPN patients showed more difficulties in glycolysis metabolizing during activation compared to those from HSP patients without nephritis, as indicated by a further reduced S6 phosphorylation (Fig. 4e).

## Discussion

Our study discovered that the decreased glycolysis rate introduced NK cell dysfunction in HSP patients. HSP is the most common systemic small-vessel vasculitis in childhood, which is mediated by an antigen-stimulated increase in levels of IgA and deposition of IgA-containing immune complexes [5]. Generally, HSP is self-limited, but there are severe complications, one of which is nephritis. The long-term morbidity of HSP is related to nephritis [4]. Given that HSP occurs more common in winter, autumn, spring, and most patients got infective trigger before the onset of disease, it is hypothesized that infections play important roles in the etiology of HSP [6, 40].

NK cells have crucial roles in protective immunity against tumors and pathogen-infected cells. The disorder of NK cells regulation may cause the obstacle of removing foreign antigens and generate the immune injury [41]. Consistent with previous studies, we found that the frequency of NK cells in peripheral blood were reduced in the HSP patients [34]. Our data also showed the activation, cytotoxicity and metabolic reprogramming of HSP NK cells were aberrant, which means the impaired immunosurveillance of NK cells. The pathogens infection may trigger or exacerbate the process of HSP or HSPN [6]. It has been reported that the N-acetylgalactosamine (GalNAc) on the surface of pathogens may facilitate the production of galactose-deficient IgA1, which are prone to aggregate into macromolecular complexes [19]. For instance, *Helicobacter pylori* infection increases the risk of HSPN, probably because of the promotion of IgA1 synthesis [42, 43]. Additionally, a positive throat culture for group A *Streptococcus* has been found in 20–30% of HSP patients [44]. In particular, based on the relevant studies and case reports, a variety of virus such as human parvovirus B19 and hepatitis B virus are also associated with trigger of HSP [6]. On the other hand, NK cells also participate in the synthesis of IgA from B cells directly [21, 45]. Possibly, these may affect the onset and progress of HSP and highlight the potential relationship between NK cells and immune injury of kidney.

Our results showed that HSPN group showed more severe disorders, which was reflected in less percentage of NK cells, lower expression of NKp46, and lower glucose uptake capacity, compared with HSP patients without nephritis. Following the progress of disease, there is a chance that HSP progress to HSPN. In consider of the clinical manifestation, HSPN can be regarded as more severe disease compared with HSP patients that only present skin involvement. The abnormal microenvironment, such as the elevated IgA or decrease IL-2, may contribute to the dysfunction of NK cells [22, 23, 34, 46]. The reason is that IgA is able to bind to Fc receptors of NK cells and mediate the inhibition of cytotoxic effect [22, 23]. IL-2 is an important cytokine for immune cells and responses, probably contributing to the reduction of Treg cells in HSP patients [14, 47]. Glomerular deposition of a streptococcal antigen may be responsible for some of the cases of HSPN [48]. However, we compared HSP patients accompanied arthritis or gastrointestinal involvement with those only present skin involvement, there were no significant difference in NK frequencies, activation and cytotoxicity (data not shown). It suggests that the nephropathy may contribute to NK dysfunction in HSPN patients. Especially, NK cells constitute a large fraction of total lymphocytes in healthy human kidneys (25% of lymphocytes) and are involved in acute kidney injury, chronic kidney disease even kidney allograft rejection [49, 50]. These show crucial roles of NK cells function in nephropathy. Therefore, the severity of the disease and kidney involvement are probably responsible for the more serious dysfunction of NK cells in HSPN.

Additionally, several studies discovered the excessive inflammatory response of HSPN patients [51, 52]. It has been reported that the apoptosis inhibitor of macrophage (AIM) was elevated in HSPN group and IL-8, IL-10, and TNF-α were positively correlated with the urinary protein levels [51, 52]. Among these cytokines, TNF-α was significantly higher than that of HSP patients without nephritis [52].

The cellular metabolism of NK cells is important in immune cells responses. Previous studies on metabolic regulation of lymphocyte mainly focused on T lymphocytes, while recent studies revealed that NK cells turned on the glucose-driven glycolysis and OXPHOS upon activation [25–27]. The glycolysis, as a dominant form of metabolism reprogramming, was proved critical for NK cell cytotoxicity [25, 30]. Indeed, aerobic glycolysis is adopted by various types of cells engaging in robust growth and proliferation as glycolysis generate energy rapidly and provide the biosynthetic precursors [37]. Several studies demonstrated that the dysregulation of glycolysis limited NK cells responses in a number of pathological conditions, including obesity and virus infection [31–33]. In fact, NK cells exhibited increased metabolism in obese children [32], while in obese adults, the lipid accumulation negatively impacted NK cells metabolism [31]. The internal mechanism is undefined but it is certain that the aberrant metabolism, whatever excessive or deficient status, will contribute to NK cell dysfunction [31, 32]. In addition, the disorder of NK cells glycolysis in obesity may lead to an overall inflammatory environment [53, 54]. It was found recently that NK cells exhibited enhanced glycolysis rates in murine inflammatory bowel diseases model [55]. These studies highlighted the probability that the dysfunction of NK cells may be involved in the symptoms of inflammation in HSP patients.

The metabolic reprogramming ensures NK cells possess the energy and biological intermediates required for producing effector molecules, such as lytic granules and cytokines [36]. Our data showed the glycolysis rate of activated NK cells from HSP and HSPN group were decreased compared with that from HC group, accompanied with an attenuated NK cells cytotoxicity. Consistently, other studies also found the expression levels of effector molecules were down-regulated when the glycolysis rates of NK cells were inhibited [31]. Indeed, glycolysis rates have been linked to the production of IFN-γ because glycolytic enzymes, GAPDH, can directly modulate the translation of IFN-γ mRNA [56]. However, the exact mechanism linking glycolysis to the expression of these effector molecules still needs further study. Although recent studies emphasized the importance of glycolysis in CD107a expression of NK cells, we did not found significant difference of CD107a expression between HSP patients and healthy controls [57, 58].

The metabolism of NK cell is highly dependent on mTORC1, a complex functioning as a nutrient and metabolic sensor and coordinating protein synthesis [39]. The mTORC1 activity of NK cells were shown to elevate upon cytokine stimulation, promoting glycolytic flux enhancement [26, 27, 59]. Our data demonstrated that NK cells from HSP patients possessed a lower mTORC1 activity than that from healthy children upon cytokine stimulation, in line with the decreased NK cell glycolysis rate in HSP patients based on our study. Beyond mTOR signaling pathway, sterol regulatory element-binding protein (SREBP) and cMyc have been shown importance in NK cell metabolism [60, 61].

Overall, our study demonstrated the deficiency of NK cell activation and cytotoxicity in HSP patients. NK cells may play a critical role in the onset and progress of HSP and HSPN. For the first time, we discovered that the dysfunction of NK cells from HSP patients was correlated with the decreased glycolysis rate, suggesting that metabolic reprogramming of NK cells may be involved in the pathogenesis of HSP. The study enhances the understanding of HSP pathogenesis. The dysfunction of NK cells probably indicated the increased risk of nephritis, which may improve the prediction of patients with HSPN. Additionally, the deficiency of NK cell activation and cytotoxicity in HSP remind us to pay more attention to the impaired immunosurveillance and the disorder immune response.

## Conclusions

Overall, we discovered the dysfunction of NK cells in HSP patients, which was accompanied by a decreased glycolysis rate. Compared with HSP patients, HSPN patients showed more severe disorders in NK cells frequencies, activation and glycolysis. It suggested that metabolic reprogramming of NK cells may be involved in the pathogenesis of HSP and highlighted the potential relationship between NK cells and immune injury of kidney.

## Methods

### Patients

A total of 64 children with acute HSP were enrolled from November 2019 to July 2020 at Beijing Children’s Hospital. Children have been diagnosed as Henoch-Schonlein purpura according to EULAR/PRINTO/PRES criteria for Henoch-Schonlein purpura [62]. All of them have not been administered with glucocorticoids, immunosuppressive drugs or heparin in the 4 weeks prior to disease occurrence. Depending on the presence of renal damage, 64 cases were divided into two groups, HSP without nephritis and HSP with nephritis. Additionally, 34 children under regular physical examination were recruited as the healthy control group. The information of the patients were displayed in Table 1. The study was approved by the Medical Ethics Committee of Beijing Children’s Hospital, Capital Medical University. Written consents were provided by all participants and their parents or legal guardians. Peripheral blood samples were collected in BD Vacutainer™ plastic blood collection tubes with EDTA K2 as anticoagulant at the onset of the disease.

### PBMC isolation and NK cell enrichment

For PBMC isolation, freshly EDTA anticoagulated blood was diluted with PBS solution and layered carefully on Ficoll-Hypaque density gradients. After centrifuged at 1000 g for 20 min at room temperature (RT), interphase cell layer was carefully transferred into a 15 ml tube. The cell pellet was washed with 10 ml PBS and centrifuged at 600 g for 5 min. The viability of isolated PBMC was determined by trypan blue exclusion staining. For enrichment of NK cells, 50 μl/mL RosetteSep™ Cocktail (STEMCELL Technologies, Canada) was added into EDTA anticoagulated blood. Mix and incubate at RT for 20 mins. Then the NK cells were collected following PBMC isolation procedures. The purity of enriched NK cell was detected by flow cytometry.

### Flow cytometry

For surface marker labeling, PBMC or NK cells were re-suspended in PBS containing 5% FBS and incubated with indicated antibodies for 30 min in dark. Wash samples with PBS before detection. For intracellular labeling, NK cells were permeabilized using Fixation/Permeabilization Solution Kit (BD Biosciences, USA) according to the manufacturer’s protocol after surface marker labeling. Flow cytometry data of stained cells were acquired with LSRFortessa flow cytometer (BD Biosciences, USA). Data were analyzed with FlowJo (v10, Tree Star). Following antibodies were used: CD56 FITC, CD56 APC, CD3 APC-Cy7, Glut-1 FITC, CD16 PE, NKp30 PE, NKp46 PerCP-Cy5.5, CD107A APC, Perforin PE, IFN-γ APC, granzyme B PE-Cy7. All antibodies were purchased from BioLegend.

### NK cell glucose consumption analysis

PBMC or isolated NK cells were washed and transferred to glucose-free media (Gibco, Invitrogen, USA) with 2-NBDG (Life Technologies, USA), 100 μM. After 30 mins culturing in dark, cells were transferred to ice immediately and stained with indicated antibodies. To activate NK cells, IL-15 (10 ng/ml), IL-2 (20 ng/ml) and IL-12 (30 ng/ml) were subjected for 18 h. Glucose uptake were determined with flow cytometry.

### Lactate release assay

NK cells were seeded into 48-well culture plate, 4 × 10^5^ cells in 500 μl medium per well. The culture medium contains IL-15 (10 ng/ml), IL-2 (20 ng/ml), and IL-12 (30 ng/ml). The supernatant (100 μl) from each group were collected after 18 h culturing. Centrifuge at 200×g, 5 min to get rid of existing cells. The cell-free supernatant were subjected to lactate assay with an EnzyChrome L-lactate Assay Kit (ECLC-100) following the manufacturer’s instructions (BioAssay Systems, USA). Briefly, add samples from different groups into 96-well plate, 20 μl per well. For each sample and standard well, working reagent was prepared by mixing 60 μl Assay Buffer, 1 μl Enzyme A, 1 μl Enzyme B, 10 μl NAD and 14 μl MTT. No Enzyme A control. Then 80 μl working reagent was added into each well quickly. After incubation for 20 min at RT, samples were measured with OD 565 nm. The L-lactate concentration of samples were calculated from the standard curve.

### Cytotoxicity assay

NK cell-mediated cytotoxicity against K562 cell lines was assessed using the CytoTox 96® Non-Radioactive Cytotoxicity Assay (Promega, USA), according to the manufacturer’s protocol. Briefly, NK cells (2 × 10^5^/well) and K562 cells (2 × 10^4^/well) were seeded into round-bottom 96-well culture plate, at a ratio of 10:1. Only NK cells and only K562 cells were seeded as spontaneous LDH release control for effector cells and target cells respectively. Cells were centrifuged at 250×g for 4 min at 20 °C after incubation at 37 °C for 6 h. The lysis solution was added to the target cell control wells 45 min prior to supernatant harvest for determination of maximal LDH release. Then, a total of 50 μl supernatant from each well was transferred to a flat-bottom 96-well plate. 50 μl/well reconstituted substrate mix were added and incubation at room temperature in dark for 30 min. Then 50 μl stop solution was added and absorbance at 490 nm was read by TriStar2 LB 942 Multimode Reader (Berthold, Germany). The cytotoxicity of effector NK cells to target cells was calculated as follows: [(Experimental effector spontaneous-target spontaneous)/(target maximal-target spontaneous)] × 100.

### Elisa

Isolated NK cells were seeded into 48-well culture plate, 4 × 10^5^ cells in 500 μl medium per well. The culture medium contains IL-15 (10 ng/ml), IL-2 (20 ng/ml), and IL-12 (30 ng/ml). After 18 h culturing, supernatants were collected and IFN-γ, perforin were quantified with ELISA kit (Invitrogen, USA) following the manufacturer’s instructions. Briefly, 20 μl serum of each sample or standard and 80 μl sample diluent were added to the wells after pre-wash the plate. Then 50 μl Biotin-Conjugate was added and incubated at RT for 2 h. Wash the plate and added 100 μl streptavidin-HRP to all wells, incubated at RT for 1 h. Then wash the plate and added 100 μl substrate and incubated at RT for 30 min in dark. Added stop solution and results were calculated by OD 450.

### Statistical analysis

The data were represented as the mean ± SEM. Statistical analyses were performed using one-way ANOVA and Tukey’s multiple comparisons test or two-tailed Student *t*-tests (unpaired) in Prism 7.0 (GraphPad Software, USA). Significant differences between groups are represented by *p < 0.05, **p < 0.01, and ***p < 0.001.

## Data Availability

The datasets used and/or analysed during the current study are available from the corresponding author on reasonable request.

## Abbreviations

HSP: Henoch-Schonlein purpura
HSPN: Henoch-Schonlein purpura nephritis
NK: Natural killer
TGF-α: Transforming growth factor alpha
Th1/2/17: T helper type1/2/17
Treg: Regulatory T cell
OXPHOS: Oxidative phosphorylation
IFN-γ: Interferon gamma
NKR: NK cell receptors
2-NBDG: 2-Deoxy-2-[(7-nitro-2,1,3-benzoxadiazol-4-yl)amino]-D-glucose
mTOR: Mammalian target of rapamycin
mTORC1: Mammalian target of rapamycin complex1
